# Transplacental neurotoxicity of cypermethrin induced astrogliosis, microgliosis and depletion of let-7 miRNAs expression in the developing rat cerebral cortex

**DOI:** 10.1016/j.toxrep.2020.11.007

**Published:** 2020-11-24

**Authors:** Imam Hassouna

**Affiliations:** Physiology Unit, Zoology Department, Faculty of Science, Menoufia University, Shebin Elkom, Egypt

**Keywords:** Cypermethrin, Neurotoxicity, RAPD, Astrocytes, Microglia, let-7 miRNAs, Rat brain cortex

## Abstract

The use of type II pyrethroids, cypermethrin is becoming a growing concern among environmental research centers. While most studies have attempted to cover the areas of DNA damage and microglia activation following exposure to cypermethin in the adult or postnatal life, less is known about the exact degree of neurotoxicity that results from exposure to transplacental sublethal doses of cypermethrin. To study the transplacental neurotoxicity of cypermethrin, pregnant rats were orally administered 10 % of LD_50_ (25 mg/kg body weight) cypermethrin, one dose daily for one week during the gestational days 15–21. The pups were investigated at postnatal day7, 14 and 21 after birth. In brain, DNA alterations were detected, astrocytes and microglia quantification were performed and some let7 family member miRNAs are estimated. The results show a gain of three major bands in the range of 350bp to 2100bp with high intensities in cortex exposed to cypermethrin compared with similar pattern indicating unaffected genomic regions in thalamus and hypothalamus at 21days. Moreover, increases in the percentage of GFAP positive astrocytes and IBA1 positive microglia indicate astrogliosis and microgliosis respectively due to cypermethrin treatment in cerebral cortex. For the first time, drastically reduced expression of let7a, b and c members are also associated with gliosis and DNA alterations, which are detected in cerebral cortex, following transplacental neurotoxicity of cypermethrin. Taking together, these results suggest that cypermethrin neurotoxicity may be mediated partly through let7 miRNAs.

## Highlights

• Transplacental neurotoxicity of the pyrethroid insecticide, cypermethrin
• DNA alterations and immunohistochemical staining of astrocytes and microglia
• Cypermethrin induces astrogliosis and microgliosis in cerebral cortex
• MicroRNAs let7a, b, and c deplete in cerebral cortex of rat pups at postanal days

## Graphical abstract

**Figure:**
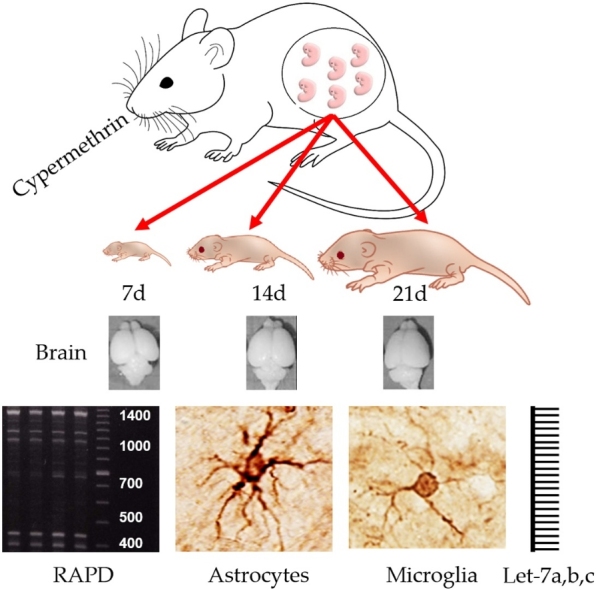

## 1 Introduction

According to their toxicity syndromes in rodents, pyrethroids are divided into two types. Type I pyrethroids produce tremor (T) and type II pyrethroids causing choreoathetosis, and profuse salivation (CS). Chemically, cypermethrin belongs to the CS type containing an alpha cyano group on its alcohol moiety [[1], [2], [3]]. Because of the broad use of cypermethrin in medical, agricultural and veterinary applications, scientists and research centers have become increasingly concerned about its possible hazardous effects towards humans [4,5].

Molecular and cytogenetic methods have been used to estimate the DNA damaging potential of cypermethrin. According to an in vivo study, in which cypermethrin was given to pregnant rats, the alkaline comet assay was not able to detect significant DNA damage from cypermethrin-treated dams in fetal blood and liver samples [6]. However, a cytogenetic study showed a substantial induction of micronucleated erythrocytes and chromosomal abnormalities in the bone marrow obtained from rats exposed to different doses of cypermethrin [7]. Additionally, cypermethrin exposure resulted in free radical-mediated brain tissue damage [8,9], leading to severe DNA damage in mice and rat brain as revealed by the comet assay [10] and DNA laddering [9] respectively. The difference in DNA damaging potential of cypermethrin might be due to several reasons such as exposure during prenatal and adult life; affected organ; dose; and rate of inactivation and elimination of cypermethrin. Furthermore, concerns have also been expressed about the usage of different DNA damage detecting methods. Compared to comet assay or DNA laddering, random amplified polymorphic DNA (RAPD) is a highly sensitive analysis. Because genomic DNA could be amplified by a single primer with arbitrary nucleotide sequence, RAPD is used extensively to detect genotoxicity/alterations including rearrangement, additions or deletion of DNA patterns and to detect DNA changes in the entire genome [11, 12]. Therefore, it is reasonable to use the RAPD technique to investigate the degree of genotoxicity produced by the cypermethrin treatment in this study.

While most studies have attempted to cover the areas of DNA damage following exposure to cypermethin in the adult or postnatal life, less is known about the exact degree of neurotoxicity that follows exposure to transplacental sublethal doses of cypermethrin. Previous studies reported that developmental brain abnormalities [13] and insignificant DNA damage in liver tissue [6] were observed in fetuses from cypermethrin-treated dams, indicating the transfer of cypermethrin from mother to fetus. Such transplacental exposure also reduced the percentage of live borne foeti, however its genotoxic effect on DNA during postnatal development of brain regions is still unknown. Therefore, this study was undertaken to investigate the effect of gestational exposure to cypermethrin on genomic alterations in brain regions of rat offspring by RAPD technique.

The lethal7 (let7) gene was originally discovered to control cell division and differentiation in C. elegans and is one of the first known microRNA in human. MiRNAs are small non-coding and single-stranded RNAs, which are capable of controlling gene expression. Research now indicates that let7 miRNA is important in several cellular processes starting from cell division, differentiation, tumor formation and suppression and implicated in the inflammation process of several diseases. Let7a miRNA was found to play a role in the microglia function during neuroinflammation, thereby its expression was significantly reduced under lipopolysaccharides (LPS) stimulation and involved in the regulation of the anti-inflammatory function of microglia [14]. Interestingly, let7b miRNA activated microglia and neurons toll-like receptor TLR7 by releasing TNFα and inducing neurodegeneration [15]. In cerebrospinal fluid obtained from patients, expression of let7b miRNA was detected with higher amounts compared with healthy people suggesting a role in mediating neurotoxicity and considered as novel biomarkers for Alzheimer's disease [16]. Recently, remarkable microgliosis was found to be associated with down regulation of let7a in the striatum of parkinsonian rats suggesting that depletion of let7a modulating the function as well as the phenotype of microglia in sustained neuroinflammation [17]. In another rat model for stroke, treatment with anti-let7 significantly reduced the infarct volume in cortex and striatum resulting in attenuation of microglia activation and neuroprotection [18]. Furthermore, increasing let7 expression antagonized brain inflammation in an in vitro ischemic stroke model [19]. Although more evidence is accumulating nowadays suggesting that the let7 miRNA family is involved in brain inflammation during neurodegenerative diseases, their expression in cypermethrin-induced neurotoxicity has not been yet investigated.

## 2 Materials and methods

Albino Wister rats of both sexes (150 g) were maintained on the 12 h dark/light cycle. One male together with two females were housed in a plastic cage. Water and rodent chow were freely available during the whole day. Two weeks after pairing, the female’s waist will start to expand if they are pregnant and they should be isolated into separate cages before parturition and during lactation. To estimate the neurotoxicity of cypermethrin on the brain, pregnant rats (five animals/group) were orally administered 10 % of LD_50_ (25 mg/kg body weight) cypermethrin (NRDC149, technical grade trans: cis; with purity of 92.4 %) daily dose dissolved in corn oil or equal volume of oil as control vehicle for one week. The administration was performed during the gestational days 15–21, a period of organogenesis, which corresponding to the second trimester of humans. The day of birth was calculated as postnatal day 1 and the pups were killed at postnatal day7 (P7), 14 (P14) and 21(P21) after birth. This treatment scheme has been used to detect the developmental effects on the nervous system by environmental chemicals. All experiments were performed according to the protocol of care and use of experimental animals approved by the corresponding faculty ethical committee.

Rat pups were anesthetized using Avertin (Tribromoethanol, Sigma-Aldrich, St Louis, MN, USA, 276 mg/kg) then sacrificed by decapitation; and immediately whole brains were rapidly removed from the skull and kept on ice for dissection. Tissue samples obtained from brain parts such as cerebral cortex, thalamus, hypothalamus, cerebellum and pons weighed and immediately homogenized by sonication in 10 vol of cold lysis buffer for DNA or RNA extractions.

### 2.1 Random amplified polymorphic DNA (RAPD) analysis

DNA was extracted from brain parts of control and cypermethrin treated pups using the isolation kit (Qiagene, Hilden, Germany). Concentrations and purity of DNA were verified by spectrophotometric analysis and ethidium bromide staining in 1.5 % agarose gels. Ready-To-Go RAPD kit (27950201), obtained from Amersham Biosciences, was used. This kit contained six (10-bp) primer sets (primer sets P1 to P6) and RAPD analysis beads with thermostable polymerases, lyophilized buffer (10 mM Tris, 30 mM KCl, and 3 mM MgCl_2_ (pH 8.3) in a 25 μl reaction volume. One set of primers was excluded due to higher annealing temperature. DNA amplification was carried out by using 25 pmol of primer, added to deionized water to a final volume of 25 μl, and 10 ng of template DNA. The primer sets were run in triplicate for each sample after optimization of the annealing temperature. The amplification was carried out in a Perkin- Elmer thermal cycler designed for 45 cycles as follows: 1 st cycle: 5 min at 95 °C, 1 min at 36 °C, 2 min at 72 °C; 44 other cycles: 1 min at 95 °C, 1 min at 36 °C, 2 min at 72 °C; followed by a last extension cycle: 15 min at 72 °C. Amplified products were uploaded on a 1.5 % agarose gel, stained by ethidium bromide and photographed under UV illumination. Data matrices were created by scoring all RAPD bands from 100 to 2,500bp. Jaccard's coefficient (J) = a / (a + b + c) was used to compare the similarity between brain parts, where a is the number of positive bands shared by both samples, b is the number of bands present only in sample 1 and c is the number of bands present in sample 2. Each band is represented as a single marker and was ascribed the value 0 or 1 indicating absence or presence respectively.

### 2.2 Quantification of let7 miRNA by RT- qPCR

Rat pups obtained from control and cypermethrin treated dams were divided into three groups (5 animals each), and decapitated at 7, 14 and 21 days postnatal. Tissues of brain areas were homogenized in lysis buffer and miRNA additive was added and kept on ice for 10 min. Total RNA was extracted from each sample according to the manufacuturer's instructions by using mirVana miRNA isolation kit (Ambion,TX). The extracted RNA was tested by gel electrophoresis; quantitated by NanoDrop spectrophotometry and immediately stored at −80 °C for later analysis. Reverse transcription kit of miRNA provided by the Applied Biosystems TaqMan was used for synthesis of cDNA. TaqMan universal PCR master mix and microRNA assays (life technologies) including primers and probes were used for the quantification of all let-7 miRNAs (let7a, let7b and let7c). Furthermore, rno-U87, *Rattus norvegicus* the small nucleolar RNA, exhibited stable expression in rat brain and considered as internal control with a primer sequence: rno-U87 forward ACAATGATGACTTATGTTTTT and reverse GCTCAGTCTTAAGATTCTCT [20]. All experiments were run in triplicate including negative control without template. Changes in mature microRNA expression were calculated according to the delta-delta CT method [21], and presented as percentage from the corresponding control at different time intervals (7, 14 and 21 days).

### 2.3 Immunohistochemical staining of GFAP and IBA1

For immunohistochemical staining of the brain, five animals per treatment group were perfused with PBS followed by cold 4% paraformaldehyde fixative. After perfusion, brains were rapidly removed from the skull, kept in paraformaldehyde for 24 h and moved into sucrose solution for 48 h. Coronal brain sections (30 μm) were cut by using cryostat and every fifth section was selected for GFAP and IBA1 staining. Sections were pretreated with an antigen retrieval solution (10 mmol/L sodium citrate buffer, pH 6.0) followed by blocking in normal goat serum to prevent unspecific binding. Then incubation with respective primary antibodies (mouse GFAP, 1:1000 Novocastra UK; rabbit IBA1 1:5000, Wako Germany) was performed for 48 h in a moist chamber. After washing three times for 15 min with PBST, sections were incubated in the appropriate biotinylated secondary antibodies for 2 h followed by washing again and ABC complex. Finally, the color was developed with diaminobenzidine (DAB) substrate solution. The slides were permanently mounted with DPX and investigated under the light microscopy (Olympus IX50). GFAP^+^ astrocytes or IBA1^+^ microglia were counted within a defined frame of the cerebral cortex in five sections per animal. The numbers obtained from both sides of the cortex were averaged for each animal and five animals were used for each treatment group to calculate the percentage differences compared with the control group.

## 3 Results

**Fig. 1:**
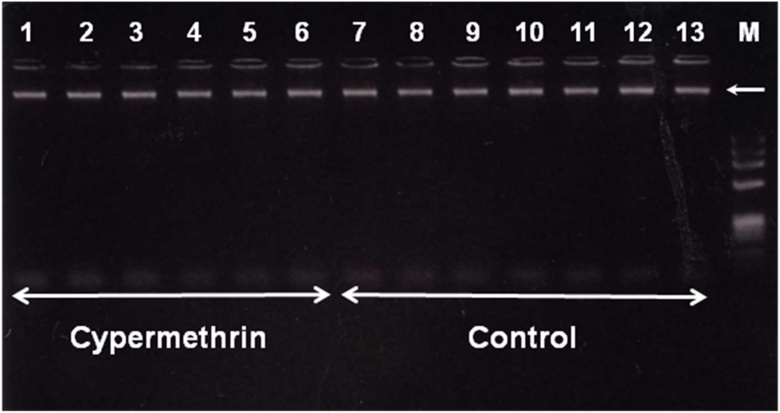
DNA from cortex of cypermethrin exposed pups (lanes from 1 to 6) and their normal counterparts (lanes 7 to 13) were extracted at postnatal day 7 (lanes 1,2,7,8); 14 (lanes 3,4,9,10); and 21 (lanes 5,6,11,12), according to the protocol described in material and methods and analyzed by electrophoresis on 1.5 % agarose gel, then visualized by ethidium bromide staining. Lane 13 shows DNA extracted from liver of control animal. Lane M shows 1 kb DNA ladder. Arrow indicates the single DNA band with higher molecular weight and without any kind of degradation or fragmentation.

To study the effect of cypermethrin on the DNA alteration, RAPD-PCR assay was carried out using extracted DNA from brain parts of control and cypermethrin treated animals. Fig. 1 shows representative photographs of DNA samples obtained from different brain parts and liver of control and treated animals indicating that our method for tissue homogenization and DNA extraction produced highly purified, large size and single DNA band without degradation.

**Fig. 2:**
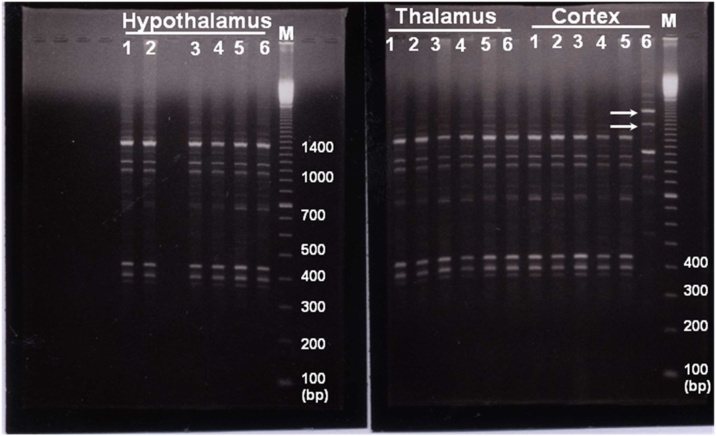
Representative RAPD patterns generated from the genomic DNA of cypermethrin-exposed brain parts at postnatal day 7 (lanes 1 and 2), 14 (lanes 3 and 4) and 21 (lanes 5 and 6) respectively. This RAPD pattern is produced by using primer (P2) showing affected genomic region in cortex of cypermethrin treated pups 21 days postnatal compared with thalamus and hypothalamus. Arrows indicate appearance of new bands above at 1600 and 2000 bp (cortex, lane 6). Lane M shows 100 bp DNA ladder. The molecular sizes (bp) are indicated on the right of each gel.

In fact, the genomic instability among the individuals of the same species must be estimated before genotoxicity studies are started. According to the investigation of RAPD pattern, there was a higher degree of reproducibility of RAPD profiles in control and cypermethrin treated animals. In all the cases, genetic instability were due to the absence and presence of the amplified bands in the cypermethrin treated samples compared with the control patterns for cortex, thalamus and hypothalamus as seen in Fig. 2. A multiband profile was observed for all the primers and new RAPD bands were detected in cortex and cerebellum only at later time interval of brain development (21days) but not at early time points (7 and 14 days) for different primers. The molecular size of these bands amplified by using primer (P3), are in the range of 350bp to 2100bp.

**Fig. 3:**
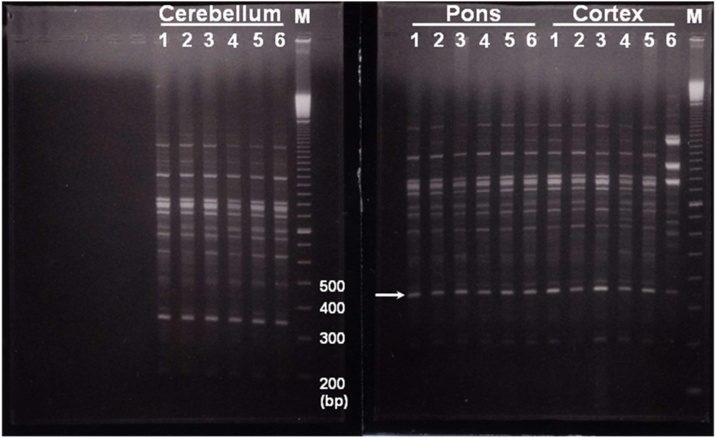
Representative RAPD patterns generated from the genomic DNA of cypermethrin-exposed brain parts at postnatal day 7 (lanes 1 and 2), 14 (lanes 3 and 4) and 21 (lanes 5 and 6) respectively. This RAPD pattern is produced by using primer (P3) showing affected genomic region in cortex of cypermethrin treated pups 21 days postnatal compared with pons and cerebellum. Arrow indicates a major band at approximately 350bp generated in all brain parts including the affected cypermethrin treated cortex (lane 6). Lane M shows 100bp DNA ladder. The molecular sizes are indicated as numbers of base pairs.

The presence of DNA damage between or within the primer's binding site will change the DNA fingerprint. Actually, several types of DNA lesions may cause the same kind of changes in RAPD profiles (e.g. decrease or increase in band intensity, as well as gain/loss of RAPD band). In cypermethrin treated pups, as shown in Fig. 3, a major band at approximately 350bp was detected in all brain samples of cortex, pons and cerebellum with the same intensity. Furthermore, three major bigger-sized bands are also observed with high intensity only in cortex after 21 days (Fig. 3, cortex, lane 6). The concentration of the template sequence is really proportional to the intensity of the amplified product. However, the presence of the major band that did not change at 350bp in all the treatment groups suggest that the increase in band intensity is not an artifact in banding pattern and may be related to cypermethrin exposure and cortex-specific.

**Fig. 4:**
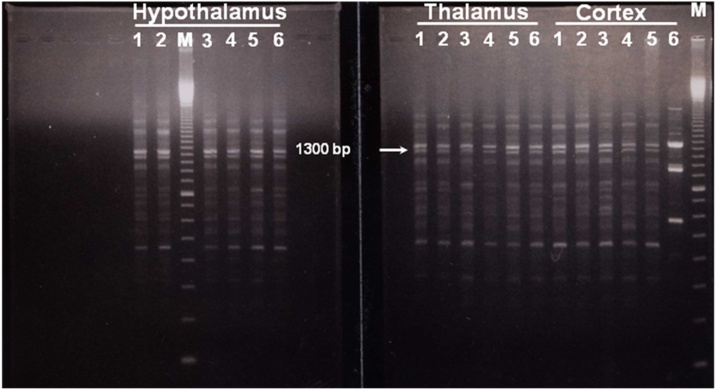
Representative RAPD patterns generated from the genomic DNA of cypermethrin-exposed brain parts at postnatal day 7 (lanes 1 and 2), 14 (lanes 3 and 4) and 21 (lanes 5 and 6) respectively. This RAPD pattern is produced by using primer, (P5) showing affected genomic region in cortex of cypermethrin treated pups 21 days postnatal. Arrow indicates a major band at approximately 1300bp generated in all brain parts, however this band is highly intensified in the affected cypermethrin treated cortex (lane 6). Lane M shows 100bp DNA ladder.

Similar results are also observed and obviously clear during the amplification of the third primer. Cypermethrin induced RAPD fingerprints showing a similar pattern indicating affected genomic regions in cortex after 21 days. A gain of three major bands with high intensities was seen in cortex exposed to cypermethrin compared with thalamus and hypothalamus (Fig. 4, cortex, lane 6). No apparent changes in the RAPD profiles were detected in cortex at 7 or 14 days. Thus, it seems likely that bands with higher intensity may be considered as diagnostic RAPD marker for cypermethrin genotoxicity in cortex after 21 days.

To verify the cypermethrin-induced genomic instability, data obtained from RAPD analysis were calculated to estimate the genomic dissimilarity index. Only five primers out of ten were chosen based on the consistency of the amplified bands and they were employed in the subsequent amplification of DNA from each of brain parts. Particularly, data obtained from the cortex and generated by the five primers (P1 to P5) are shown in Table 1. For example in cortex of cypermethrin-treated pups, the selected primers generated 30 bands, including 9 positive and 21 negative unique markers. The positive markers are only present in cortex at 21days postnatal and absent in all other brain samples, while negative unique markers are amplified in all other brain samples including controls except the cortex at 21days (Table 1). The presence of 9 positive unique makers in cortex after 21 days reflects how much the genetic background of this brain area is different from other brain parts demonstrating a genetic instability between brain parts of cypermethrin exposed pups.

**Table 1:** Cortex-specific markers obtained from the RAPD analysis.

Samples	Positive unique markers (bp)					Negative unique markers (bp)				
	P1	P2	P3	P4	P5	P1	P2	P3	P4	P5
Cortex	950, 580	2000, 1100, 950	600	2000	2500, 700	1300, 1200, 1050, 825, 600	1600, 1150, 1050, 800, 450, 400	1800, 1300, 1000, 900, 470	1050, 650	1100, 750, 650
Total	9					21				

To confirm these results, dissimilarity index is calculated to estimate the level of variation among all pairs of the brain parts using the Jaccard coefficient. For calculation, each band of the electrophoretic profiles represented a single marker and was ascribed the value 0 or 1 indicating absence or presence respectively. Table 2 shows the degree of variability among each pair of the studied brain samples with values of genetic diversity ranging from 0.14 to 0.48 using the Jaccard coefficient. The highest dissimilarity level among the brain parts was 0.48 demonstrating the large divergence between the cortex and cerebellum. While the lowest dissimilarity index was 0.14 between hypothalamus and thalamus indicating that, they are approximately 90 % genetically identical.

**Table 2:** Dissimilarity index among the five brain parts using Jaccard similarity coefficient.

	Cortex	Hypothalamus	Thalamus	Pons	Cerebellum
Cortex	0.00				
Hypothalamus	0.31	0.00			
Thalamus	0.34	0.14	0.00		
Pons	0.34	0.23	0.23	0.00	
Cerebellum	0.48	0.24	0.26	0.21	0.00

**Fig. 5:**
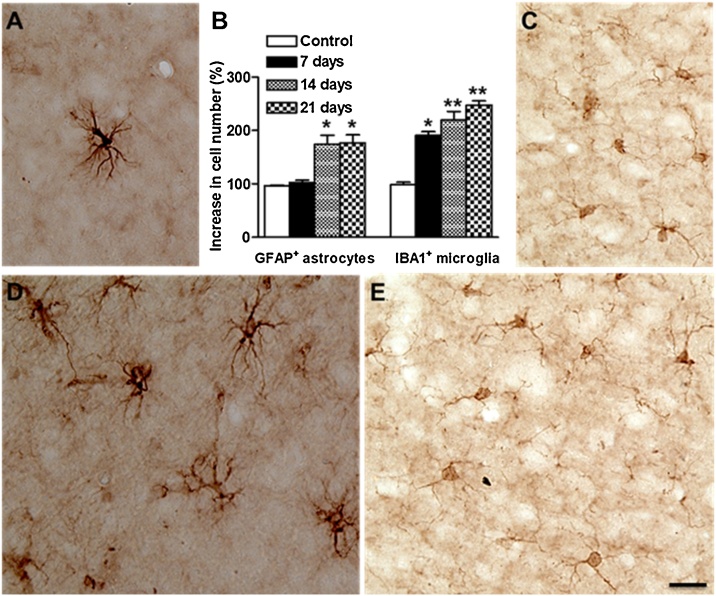
Cypermethrin effects on astrocytes and microglia in cerebral cortex of rat pups. Changes in the total number of astrocytes (A, D) and microglia (C, E) in cerebral cortex of control (A, C) and 21 days after prenatal exposure to cypermethrin (D, E). Numbers of GFAP^+^ astrocytes and IBA1^+^ microglia were counted at different time intervals after birth (7, 14 and 21 days) for control and cypermethrin treated groups and the quantitative analysis is shown in B as mean + SD. Values for each time point are normalized to the corresponding control (100 %). Statistical significance was calculated by using Student's t test, **: p < 0.001 and *: p < 0.005. Scale bar = 20 μm.

Furthermore, in order to correlate and confirm the alterations in DNA with the inflammatory conditions, changes in the numbers of reactive microglia and astrocytes as well as the expression of let7 miRNAs were quantified in cerebral cortex of pups born from cypermethrin-treated and control dams. Microglial cells have been shown to regulate brain homeostasis, neuronal differentiation and synapse formation, while astrocytes are establishing blood-brain barrier. Brain sections containing the cerebral cortex were immunostained with IBA1 (ionized calcium binding adaptor molecule 1) or GFAP (glial fibrillary acidic protein) antibodies, the most common markers for microglia and astrocytes respectively. In the control rat pups, the numbers of GFAP and IBA1 positive cells are increasing gradually with time, suggesting the glial development and maturation during the second and third postnatal week. On the other hand, cypermethrin-treated pups exhibited highly significant increases in the number of IBA1 positive cells in the cerebral cortex compared with controls, indicating microgliosis (Fig. 5C and F). These increases were observed at 7, 14 and 21 days postnatal (Fig. 5B). Similar results have been found regarding the GFAP positive astrocytes. In both sides of cortex, significant increases in the number of GFAP astrocytes were calculated only at 14 and 21 days (Fig. 5A, B and D). These data suggest that the increases in the number of GFAP and IBA1 positive cells indicating astrogliosis and microgliosis respectively due to cypermethrin treatment in cerebral cortex.

**Fig. 6:**
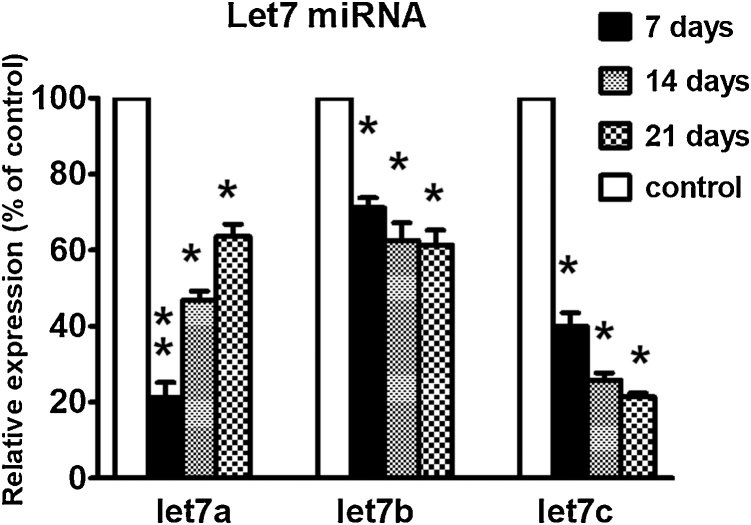
Relative expression of let7a, b and c miRNAs in the cerebral cortex obtained from three control and three cypermethrin-treated groups of pups at 7, 14 and 21days postnatal. Values for each time point are normalized to the corresponding control at 7, 14 and 21 days (100 %). Data are expressed as percentage of control ± SE. Statistical significance was calculated by using Student's t test, **: p < 0.001 and *: p < 0.005.

This study also determined the expression of let7 miRNAs in the cerebral cortex of cypermethrin-treated and control pups during postnatal developmental stages. The expression of let7 generally was higher in cerebral cortex in comparison with other brain parts. In addition, let7 expression was also increased gradually with age; i.e levels of 21 days were higher than 7 days postnatal correlating with brain development and therefore for each time point there was a corresponding control group used for normalization. Cypermethrin-treated pups displayed significant decreases in all let7 members at all time intervals examined, suggesting that down-regulation of let7 miRNA may be a marker of neurotoxicity and/or involved in the mechanism of cypermethrin-induced toxicity. In particular, let7a and c were severely affected by cypermethrin treatment (Fig. 6). Interestingly, depletion in the levels of let7a attenuated by time and reaching more than 60 % of control level at 21 days postnatal. This recovery was not observed in the case of let7b and c indicating that both members need longer time to return to normal levels.

## 4 Discussion

To investigate the genomic instability after cypermethrin exposure, RAPD patterns produced by different primers showing affected genomic regions in brain areas were performed, allowing for easy identification of multiple genomic alterations such as amplification, deletion or rearrangement, DNA damage and mutations simultaneously without prior identification of nucleotide sequence and therefore is useful to estimate the genotoxicity. This study show a gain of three major bands in the range of 350bp to 2100bp with high intensities in cortex exposed to cypermethrin compared with similar pattern indicating unaffected genomic regions in thalamus and hypothalamus at 21days. Altogether, the presence of 9 positive unique makers and absence of 21 negative markers in cortex reflects the genetic instability between brain parts of cypermethrin-exposed pups and therefore confirming that the RAPD assays is able to detect DNA alteration in the entire genome. Successfully, RAPD profiles showed appearance and disappearance of bands after environmental chemical treatment in rats suggesting the presence of DNA adducts or breaks and/or mutations [22,23]. Yet so far, there is no RAPD analysis published on the genotoxicity of cypermethrin in the literature. Indeed, a potential clastogenicity and alterations in cellular DNA in vivo attributed to cypermethrin exposure in the bone marrow of rats [13]. Massive DNA fragmentations due to cypermethrin treatment was also observed as DNA laddering [9]. Instead of the parent compound, in vitro metabolites of cypermethrin have contributed to the development of DNA monoadducts and interstrand crosslinks, which may involve cytochrome P450 in the activation of cypermethrin [24]. Furthermore, administration of cypermethrin significantly upregulated the mRNA expression of 20 genes in mice brain related to DNA mismatch repair, single strand break repair and DNA synthesis [25]. Interestingly, cypermethrin is considered by the United States Environmental Protection Agency as a possible human carcinogen and its ability to reduce host defenses against cancer has been confirmed [26]. RAPD markers obtained in this study may be interpret as indicators of genomic instability specifically during initiation of malignancy in developing brain.

Developing brain is very sensitive to the exposure of environmental toxins [27]. Due to its ability to cross blood-brain barrier, which is still in the developing process, cypermethrin in vivo can damage astrocytes, impair and make the barrier more permeable to substances as compared to adults [28]. Indeed, exposure to cypermethrin during the postnatal period induced persistent functional impairment in the developing blood-brain barrier that were normalized only after 43 days [29], implicating an important role for astrocytes. Moreover, cypermethrin was found at detectable concentrations in amniotic fluid and maternal urine of pregnant rats indicating that fetus was exposed to cypermethrin as well as its metabolites affecting the fetal compartment [30]. Furthermore, cypermethrin accumulation in the brain of prenatally exposed offsprings has the ability to affect the inducibility of cerebral cytochrome P450s, which enhances the susceptibility of rats to neuron degeneration when they rechallenged during adulthood [31]. This effect is mediated by enhanced expression of MHCII and microglial activation demonstrating that prenatal exposure to cypermethrin increases neuroinflammation in offsprings [25,32]. Therefore, understanding timing and onset of reactive gliosis by investigating astrocytes and microglial cells, is crucial for controlling the detrimental effects of transplacental exposure of cypermethrin that can impart on brain during pregnancy.

The results of this study show that transplacental exposure of cypermethrin induced astrogliosis and microgliosis in cerebral cortex of treated pups. Increased numbers of astrocytes and microglial cells are compatible with reactive gliosis, consistent with disturbances in blood-brain barrier permeability and a degree of inflammation in cerebral cortex. Although cypermethrin treatment was not able to induce profound changes in the astrocytes morphologically in rat cortex [33], measurement of GFAP levels by ELISA reader showed 20 % reductions in its concentration. However, the reduction in GFAP levels is not coincided with the increased number of GFAP positive astrocytes in this study. Three points should be taken into consideration to explain this contrast, transplacental exposure of cypermethrin, age of embryo or pups during treatment and time of analysis (3 and 7 weeks in this and their study respectively). Not only astrocytes but also bone marrow cells and blood lymphocytes increased in rats offspring exposed to cypermethrin indicating an improved ability to proliferate, however no differences were found in dams exposed to cypermethrin during pregnancy [34].

Let7 miRNAs regulate gene expression and microglia functions. Actually, there is some evidence supporting the strong relationship between microglia and expression of let7 gene family members. First, significant reduction of let7a was demonstrated under LPS stimulated inflammatory conditions, indicating its role in the modulation of anti-inflammatory function of microglia [14]. Second, being a mixed immunomodulatory miRNAs, let7 gene regulating neuroinflammation under different pathologies by promoting anti or proinflammatory response [35]. Third, let7 induced inflammatory response by directly activating TLR7 in microglia, thereby playing a role in signaling pathways in the brain [36]. Fourth, intense microgliosis, sustained neuroinflammation and reduction of let7a expression were found side by side in brain of parkinsonian rats [17]. Finally yet importantly, regarding the shape of microglia, let7c overexpression converts the microglia from M1 into M2 polarization, the neuroprotective phenotype that inhibiting neuroinflammation and enhancing CNS repair [37]. Interestingly, cypermethrin not only inhibited M1 and shifted microphages to M2 type enhancing tumor metastasis [38] but also increased percentage of ameboid, multivacuolated and activated microglia leading to neuroinflammation in embryonic and adult brain [39,40]. Consisted with the previous investigations, the results of this study imply that increased IBA1 positive microglia observed in the cortex, a brain area displayed DNA alterations, associated with depletion of let7 expression are involved in the inflammatory role of microglia following cypermethrin neurotoxicity.

To the best of my knowledge, there is no information regarding cypermethrin toxicity and let7 expression in the literature. However, it is noteworthy to mention that during the revision of this paper, a recent comprehensive review article has been published to evaluate alterations in miRNAs after exposure to different pesticides that enhance certain diseases [41]. Particularly, down regulation of let7a, b and c was associated with intoxication of mercury and lead in human placenta [42], markedly reduced let7c expression was found in pons and medulla of neurotoxin, iminodipropionitrile treated rats [43]. Similar results for let7a, b and c were also detected following lipopolysaccharide-induced neuroinflammation in rat cortex [44]. Mechanistically, ethanol released let7b in microvesicles inducing TLR7 dependent neurotoxicity [45]. On the other hand, transplacental exposure to herbicide glyphosate showed upregulated let7a in the prefrontal cortex of 28 postnatal day mouse pups [46]. Certainly, an in vitro single study has revealed that cypermethrin activated microglia cells through downregulation of miR-155 promoting inflammation [38]. Therefore, the increased number of microglia in cortex, as a marker of inflammation in this study emphasize the importance of avoiding cypermethrin exposures in pregnant women.

During rat brain development namely between E10 and P28 postnatal days, members of let7c, a, and b were found to be the top 20 highly expressed miRNAs in the cerebral cortex [47] and due to increased neural plasticity, the brain during that time was more susceptible to the damaging effects of toxicants. Very recently, in vivo studies have revealed that cypermethrin traveled across the placental and blood brain barriers with concentrations enough to impair fetal growth, reduce forebrain volume and increase forebrain proliferative zone [39]. Of utmost importance, that let7a and b expression was necessary for timing and specification of cortical layering and lamination [48]. Drastically reduced expression of let7 members in this study implicate the involvement of increased microglia and DNA alterations, which detected in cerebral cortex, in the transplacental neurotoxicity of cypermethrin. Taking together that, Let 7a and c expressions were found to regulate cell proliferation, contributing to the oncogenesis and growth [49] and let7b tune cerebral cortical neurogenesis [50], bind to TLR7 inducing neurodegeneration and propagate toxicity [15], these results suggest that cypermethrin toxicity may be mediated partly through let7 miRNAs. Further studies are recommended to clarify let7-based therapy to modulate transplacental cypermethrin induced neuroinflammation and neurotoxicity.

## Funding

This research received no external funding.

## CRediT authorship contribution statement

**Imam Hassouna:** Conceptualization, Methodology, Validation, Formal analysis, Investigation, Resources, Writing - original draft, Writing - review & editing, Visualization.

## Declaration of Competing Interest

The authors declare that they have no known competing financial interests or personal relationships that could have appeared to influence the work reported in this paper.
